# Undiagnosed Bilateral Avascular Necrosis of the Femur in a Young Male Caused by COVID-19 Steroid Injections

**DOI:** 10.7759/cureus.29982

**Published:** 2022-10-06

**Authors:** Sakshi Kamani, Madhu G Lakhwani, Pratik Phansopkar

**Affiliations:** 1 Physiotherapy, Ravi Nair Physiotherapy College, Datta Meghe Institute of Medical Sciences, Wardha, IND; 2 Musculoskeletal Physiotherapy, Ravi Nair Physiotherapy College, Datta Meghe Institute of Medical Sciences, Wardha, IND

**Keywords:** rehabilitation, pain, physical therapy, femoral head, avascular necrosis (avn)

## Abstract

Osteonecrosis is a degenerative bone disease brought on by a change in subchondral blood flow and characterized by the loss of bone cellular components. Other names for it include ischemic bone necrosis, aseptic necrosis, and avascular necrosis. Long bones' epiphyses in weight-bearing joints are typically impacted. In extreme cases, a joint may completely collapse or subchondral bone may be obliterated. Avascular necrosis, which most frequently affects joints, especially the femoral head, occurs when the blood supply to the bones is diminished. In this article, we will explain the clinical case of a 40-year-old man who's been complaining about hip pain for two months. The patient went to Acharya Vinobha Bhave Rural Hospital (AVBRH) with the same complaint and underwent some tests; upon inspection, it was discovered that the patient had bilateral avascular femoral head necrosis. For avascular necrosis (AVN) of the left femoral head, the patient had core decompression surgery. Once post-operative physical therapy was initiated, the condition significantly improved, and it also served to prevent additional abnormalities. The goal of this case study is to examine the therapeutic strategies essential for treating bilateral femoral head avascular necrosis.

## Introduction

Avascular or aseptic necrosis of bone was probably first described in 1738 by Alexander Munro [1]. Osteochondritis dissecans was the name Koenig gave to this ailment when he originally described it in 1888. After being exposed to a known risk factor, such as high-dose glucocorticoid therapy or a femoral neck fracture, avascular necrosis (AVN) manifests itself one to six months later. Even when the patient is exposed to the risk factor in the future, AVN is rare. The collapse of the necrotic part of the epiphysis is caused by subchondral plate fracture, which frequently occurs within the first two years [2]. It could have a wide range of traumatic and nontraumatic origins. These causes include congenital disorders, coagulopathy, long-term use of steroids or alcohol, fractures, and dislocations. The femoral head is most usually affected by this illness. There is a roughly 4:1 sex ratio for this illness, with men being more prone than women. Men aged 25 to 44 and women aged 55 to 75 had the highest incidence. Typical risk factors for avascular necrosis of the femoral head (AVNFH) include alcoholism, steroid use, chemotherapy, immunosuppressive medications, and sickle cell anaemia [3].

The classification system was first introduced by Ficat and Arlet in 1964. From Stage 0 to Stage 4, the subsequent Ficat classification recognized five distinct stages of bone necrosis. Early phases included Stages 0 to 2, whereas late phases included Stages 3 and 4. A "silent hip," which is preclinical and pre-radiographic, is referred to as stage 0 [4]. This problem may be treated surgically or with conservative measures. Conservative therapy has occasionally produced long-lasting healing when administered experimentally, either alone or in combination. One surgical procedure that can be used to treat avascular necrosis is an osteotomy. Other surgical techniques like bone grafting, core decompression, and arthroplasty can also be used to treat AVN. The goal is to preserve the femoral head and prevent its collapse for as long as possible keeping in mind at the same time that the maximal and definitive treatment is total hip replacement, which is the ultimate and final treatment. Efforts should be made to delay the moment when arthroplasty is needed, without compromising the chance of a straight-forward hip replacement [5].

## Case presentation

We describe the incident of a 40-year-old man who was involved in an accident a year prior and visited a hospital in Mumbai as a result. No fracture was suspected, thus the man was given medication. But despite getting plenty of rest and taking medication, the patient's sensations of pain and discomfort persisted. He opted for an orthopaedic facility in Amravati since he lived there. After conducting multiple investigations, X-rays revealed bilateral avascular hip necrosis. He had to have a history of being admitted for two months to the intensive care unit during COVID-19, and his condition necessitated the infiltration of steroidal injections. He had a core decompression treatment for his right hip in Amravati and then presented to Acharya Vinobha Bhave Rural Hospital (AVBRH) complaining of a dull, painful discomfort in his left hip. He underwent the same procedure here, but for his left femoral head, and post-operative physiotherapy sessions have continued ever since.

Clinical findings

**Figure 1:**
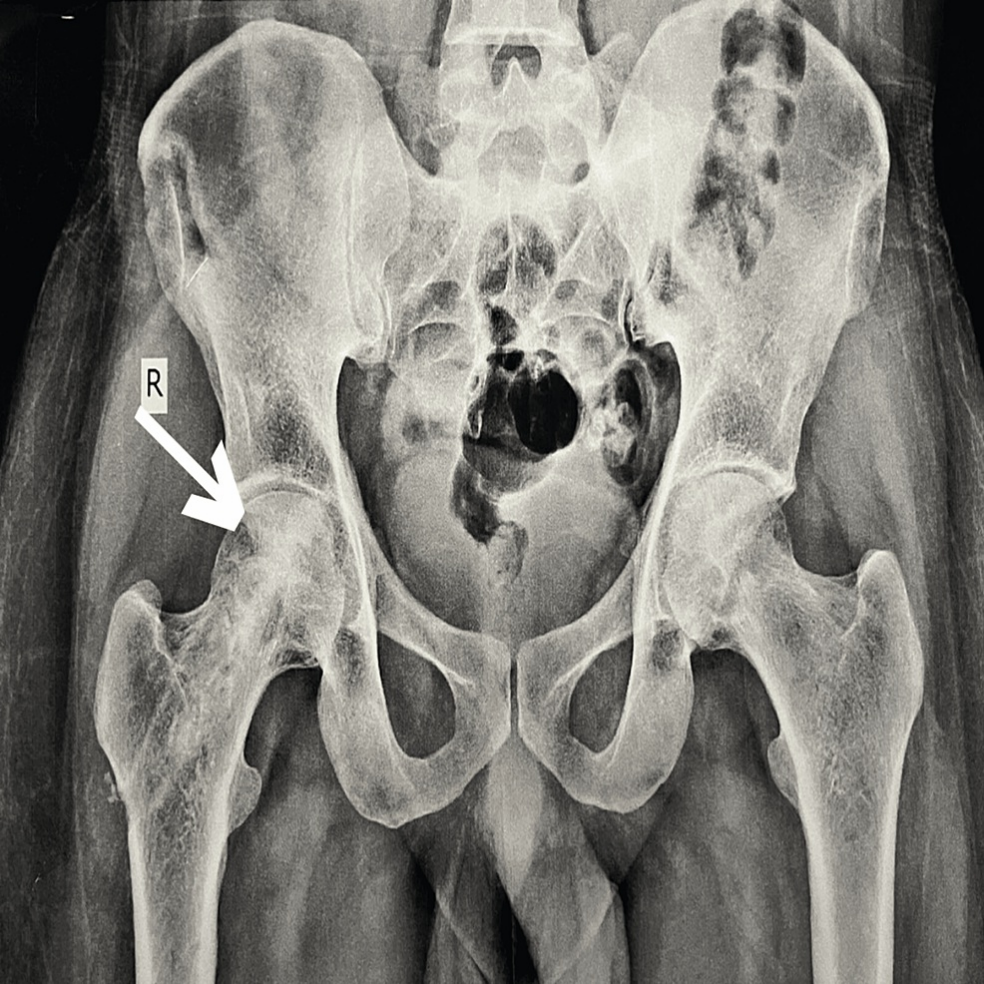
Loss of contour of right femoral head X-ray of the pelvis (anteroposterior view)

**Figure 2:**
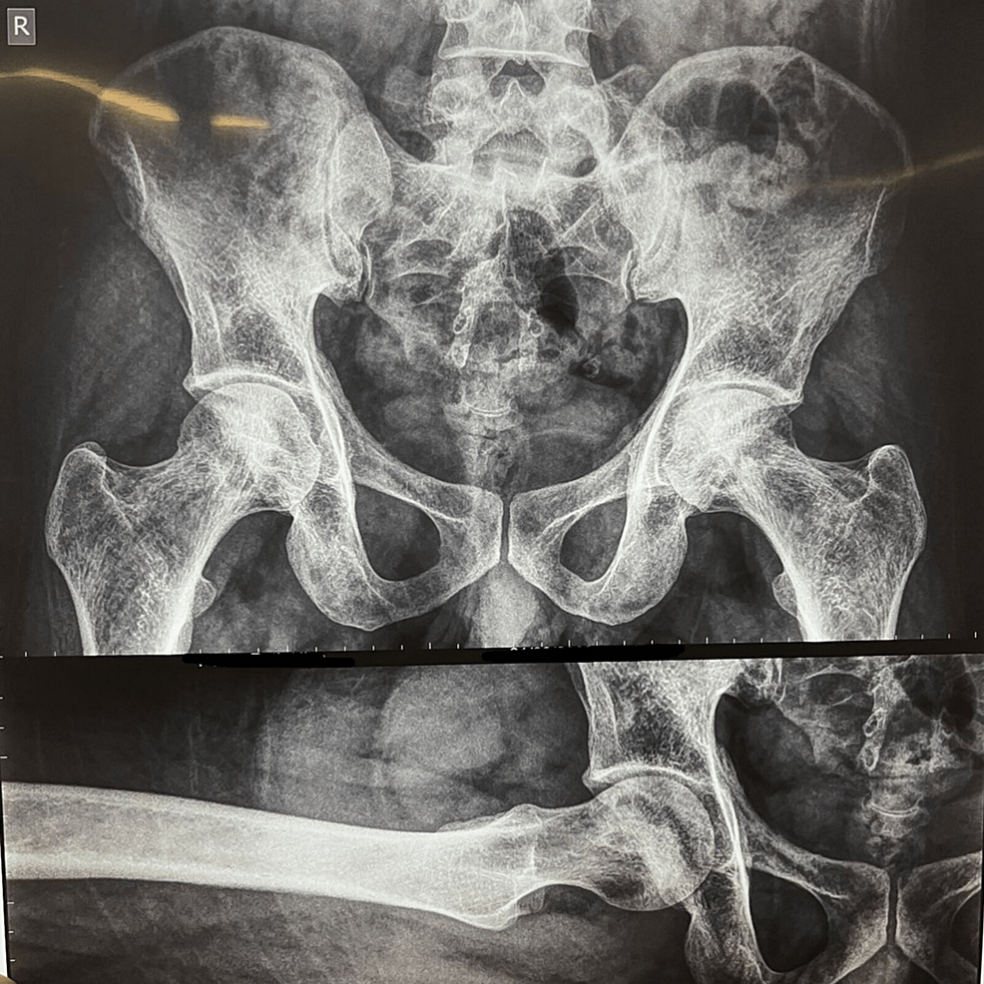
Very early degenerative changes are observed in the figure

X-ray findings reveal the pelvis with both hips in anteroposterior (AP) view in Figure 1. It also shows loss of contour of the right femoral head. Very early degenerative changes are also observed in Figure 2. MRI findings revealed stage 3/early stage 4 left and stage 2 right femoral head avascular necrosis as per modified Ficat and Arlet classifications. Also, minimal anterosuperior left femoral head collapse and mild left femoro-acetabular chondral thinning are visible.

Physiotherapy rehabilitation

On the first postoperative day (POD), a proper and systematic examination of the patient's physiotherapy was conducted. The patient was given a well-organized, six-week physiotherapy rehabilitation regimen, as indicated in Table 1. The patient was given a well-organized home programme and a follow-up schedule at the end of the three-week rehabilitation treatment in order to prevent any further issues and difficulties.

**Table 1:** Physiotherapy rehabilitation Quads - Quadriceps; Hams - Hamstrings

PHASES	GOALS	INTERVENTION
Phase 1 (Day 1 to 1 week)	Patient Education	Education regarding the condition of the patient was given to the caregivers.
	To prevent secondary complications	The patient was given ankle pumping exercises with 10 repetitions twice daily to stop thrombus development.
	To prevent post-operative complications	To prevent atelectasis or pneumonia, the patient was taught deep breathing exercises and thoracic expansion exercises.
	To improve strength	Static Quads. And Hams. with 10 repetitions twice a day.
Phase 2 (week 2 to week4)	To regain functional Range of Motion	Active movements of the unaffected extremity are continued while active assisted movements for affected extremity are started.
	To make patient functionally independent and sstable	Bed side sitting and ambulation with the help of assistive devices was continued during this phase. With continuation of phase 1 protocol, additional strengthening exercises of upper limb and lower limb (up to tolerable limit) was started with resistance band.
Phase 4 (week 4 to week 6)	To regain muscular strength and endurance	For regaining muscular strength and endurance, open chain and bilateral closed-chain exercises were performed.
	To Regain range of Motion	Complete passive range of motion in both the upper and lower extremities
	Strengthening of hip and knee extensors	The patient was instructed to perform mini squats while using light-grade elastic resistance to improve their hip and knee flexors.
	To improve postural stability	Progressive balance activities in standing were performed by the patient to improve balance and gait. In order to test his balancing system, he was also instructed to practise walking on soft, uneven terrain.

**Figure 3:**
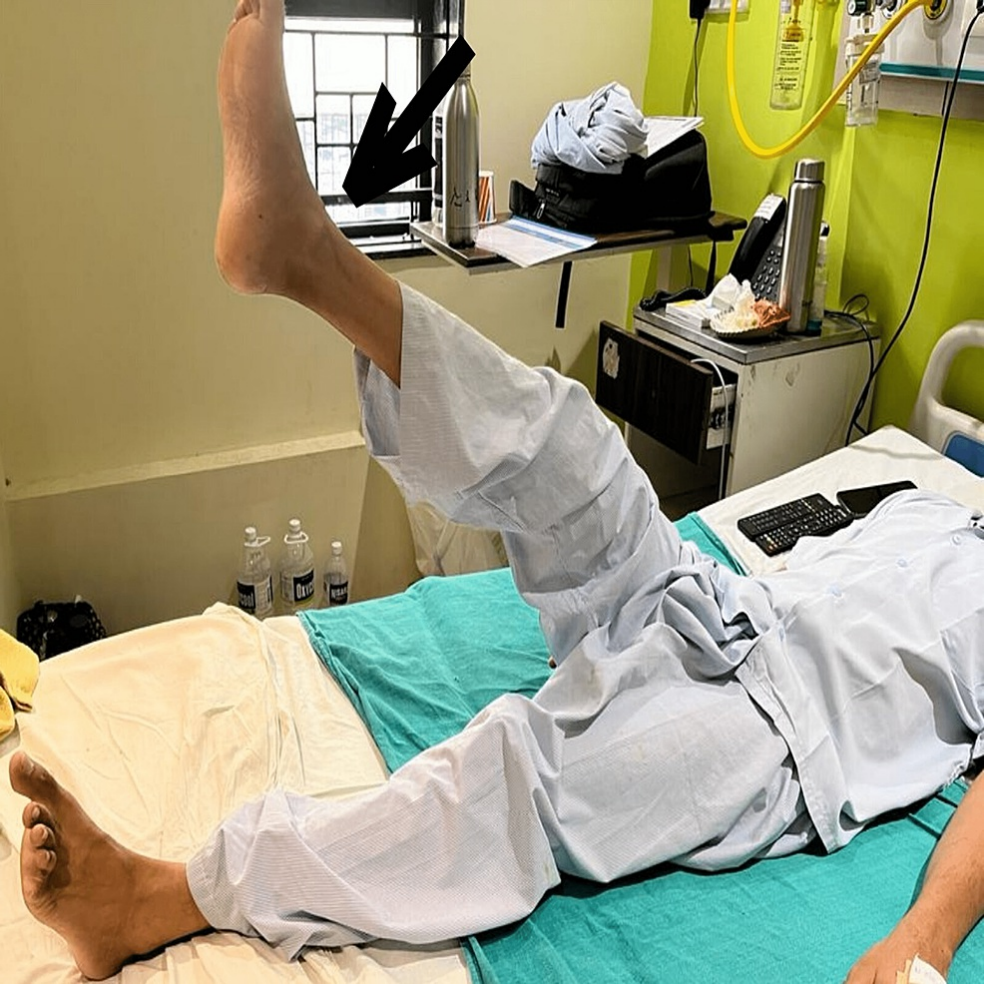
The figure shows the patient performing post-operative straight leg raise (SLR) (110⁰)

**Figure 4:**
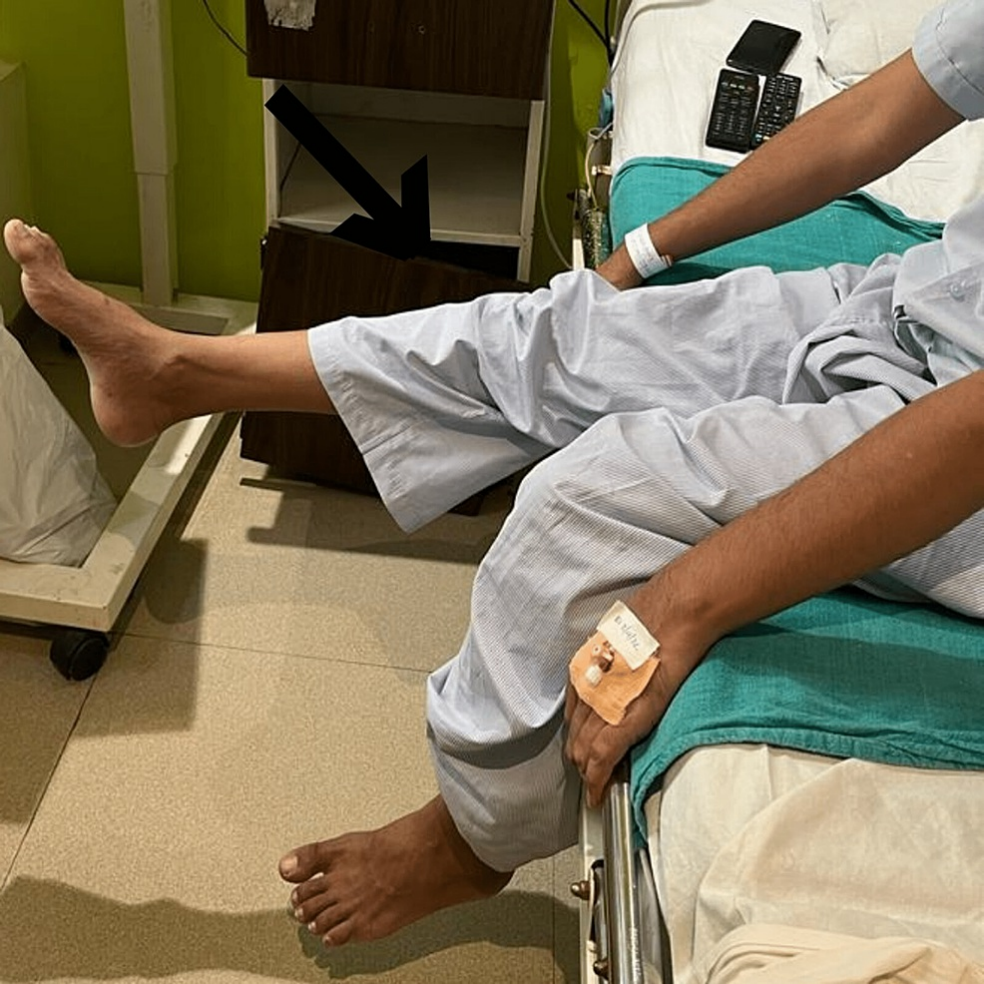
The figure demonstrates the patient performing dynamic quadricep exercise post-operatively

**Figure 5:**
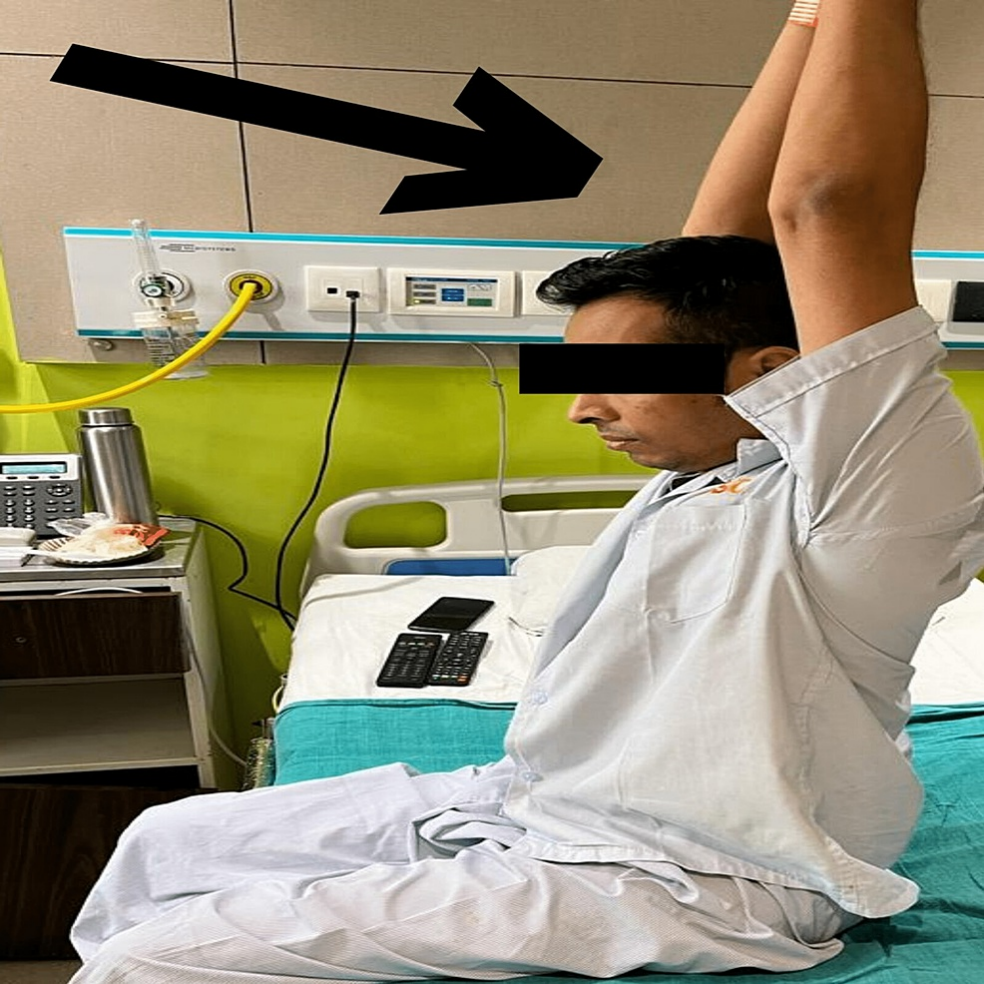
The figure shows the patient performing upper limb range-of-motion exercise post-operatively

Exercises for the patient's post-operative upper limb range of motion, dynamic quadriceps, and Straight Leg Raise are shown in Figures 3-5 which indicate a significant improvement in the patient's post-operative performance.

Table 2 lists the successful outcomes of the pre- and post-rehabilitation assessment after adhering to the rehabilitation programme. Pain on Numerical Pain Rating Scale (NPRS) was 8/10 on initial assessment and 2/10 post-rehabilitation.

**Table 2:** Pre- and post-rehabilitation assessment

Variable	Movement	Pre-op. assessment		Post-op. assessment	
		RIGHT	LEFT	RIGHT	LEFT
Range of Motion(in degrees)	Hip Flexion	0-125⁰	0-110⁰	0-160⁰	0-170⁰
	Extension	120-0⁰	115-0⁰	125-0⁰	130-0⁰
	Abduction	0-45⁰	0-45⁰	0-70⁰	70-0⁰
	Adduction	0-45⁰	0-10⁰	0-90⁰	0-80⁰
	External rotation	0-45⁰	0-40⁰	0-60⁰	0-55⁰
	Internal rotation	0-40⁰	0-5⁰	0-55⁰	0-10⁰
	Knee Flexion	0-90⁰	0-70⁰	0-90⁰	0-90⁰
	Extension	90-0⁰	70-0⁰	90-0⁰	90-0⁰
Manual Muscle Testing (Grade 0-5)	Hip Flexors	3/5	1/5	4/5	4/5
	Extensors	2/5	1/5	4/5	4/5
	Abductors	3/5	1/5	4/5	4/5
	Adductors	3/5	1/5	4/5	4/5
	Knee Flexors	4/5	4/5	5/5	5/5
	Extensors	4/5	4/5	5/5	5/5

## Discussion

Age, periodization, region of osteonecrosis, posture and collapse risk, and individual preference all factor into the therapy of avascular necrosis of the femoral head. The best therapeutic effect can only be attained by correctly grasping therapeutic principles and implementing suitable procedures specifically geared toward certain stages. Young patients who are active are frequently affected by avascular necrosis of the femoral head, which has a significant negative impact on their activity and quality of life.

Total hip arthroplasty's (THA's) long-term prognosis is yet unknown. The national health system also experiences significant financial effects from prosthetic implants in these individuals. In their trials, Calori et al. found that early on in the course of avascular necrosis of the femoral head, surgical treatment with Core Decompression (CD) combined with biotechnologies is advised [6]. Bellot et al. claim that there is debate in the literature on epidemiological factors that potentially impair outcomes following core decompression for osteonecrosis. Although early-stage disease (I or II) is thought to be the optimal indication for decompression, this is not enough to ensure success. We agree with previous writers in that Association Research Circulation Osseous Classification (ARCO) stages III and IV and a Koo index of greater than 40 are contraindications to decompression. Only by reducing indicators may core decompression outcomes be improved [7]. 

Although they have been employed experimentally alone or in combination, conservative treatments rarely result in long-lasting improvement. The therapy of avascular necrosis yields good results with both surgical and non-surgical procedures. Patients who have undergone surgery and have a well-designed physiotherapy programme recover more quickly. Bivascular necrosis of the femoral head is rare, but if treated promptly, has better outcomes. Following a six-week regimen, the patient demonstrated considerable improvement in pain relief, increased range of motion in both the upper and lower extremities, increased muscle strength, and increased functional capacity.

## Conclusions

In terms of pain relief and enhancement of upper limb motions, this case study emphasizes the value of well-structured physical therapy interventions for postoperative patients with bilateral avascular necrosis of the hip during the inpatient phase. The report concludes that the impact of steroidal injections caused by COVID-19 resulted in bilateral avascular necrosis of the femoral head in a young male, which interfered with the patient's daily activities and made it challenging for him to manage the unintended side effects of the steroidal injections. The patient's recovery was planned properly and efficiently, enabling him to become independent and capable of actively carrying out his job. Timely screening therefore helped with the condition's treatment.
